# Heat Dissipation Characteristics of IGBT Module Based on Flow-Solid Coupling

**DOI:** 10.3390/mi13040554

**Published:** 2022-03-31

**Authors:** Lipeng Tan, Peisheng Liu, Chenhui She, Pengpeng Xu, Lei Yan, Hui Quan

**Affiliations:** 1 Jiangsu Key Laboratory of ASIC Design, College of Information Science and Technology, Nantong University, Nantong 226019, China; tanlipengdsg@163.com (L.T.); 1811310001@stmail.ntu.edu.cn (C.S.); 2110320054@stmail.ntu.edu.cn (P.X.); 2110310042@stmail.ntu.edu.cn (L.Y.); 2 College of Science, Nantong University, Nantong 226019, China; 2102310022@stmail.ntu.edu.cn

**Keywords:** IGBT module, water-cooled heatsink, ANSYS, junction temperature, temperature field, fluent field

## Abstract

With the increase of power level and integration in electric vehicle controllers, the heat flux of the key silicon-based IGBT (Insulated Gate Bipolar Transistor) device has reached its physical limit. At present, third-generation semiconductor devices including SiC MOSFETs (Metal-Oxide-Semiconductor Field-Effect Transistor) are gradually replacing the dominant IGBT module. The hybrid IGBT module consists of both and can improve the performance and reduce the cost of controllers. Limits due to the installation space, location, and other conditions in the car make it difficult to meet the requirements of controllers with an air-cooled heatsink due to their large size and limited heat dissipation capacity. A smaller and more powerful water-cooled heatsink case is required to ensure the heat dissipation of the IGBT in the controller. Based on previous experience in finite element numerical simulation, hydrodynamics calculation, and heat transfer calculation, ANSYS Workbench finite element software was used to analyze the thermal resistance of each structure inside the module and the heatsink structure. The fluid characteristics and heat transfer performance of three different flow channel structures were analyzed, and the design of the cooling flow fin was improved to provide a reference for the heat dissipation of the hybrid IGBT module.

## 1. Introduction

**Figure 1:**
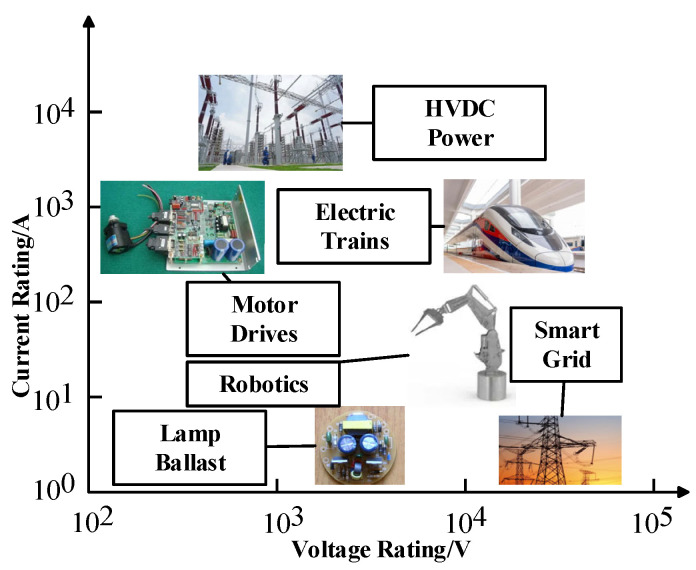
The wide spectrum of IGBT applications.

In modern society, PE (Power Electronics) systems are widely used in industrial and household applications for controlling and converting electrical energy [1]. In new energy vehicles, railways, automated manufacturing systems, wind power generation and other fields, PE systems can save energy and make structures more compact. In the past few years, power semiconductors including IGBT modules have dominated the power converter market due to their excellent performance, low cost, reliability, light weight, and small size [2,3]. One of the most important power conversion devices, IGBTs were invented in 1982, which accelerated the application of bipolar devices in high voltage and large current markets [4,5]. As shown in Figure 1, the IGBT is applied to a wide range of current and voltage levels [6]. In applications except smart grids, the rated current of IGBTs increases with the increase of rated voltage. For silicon-based IGBTs, this problem can be solved by using multi-chip power modules. A more advanced solution is to use the third-generation semiconductors, which can work at higher frequencies than silicon-based IGBTs. At present, in the application fields requiring medium and high power, the traditional low-switching frequency silicon IGBTs and the emerging high-cost SiC MOSFETs cannot simultaneously meet the performance and cost requirements of converters. While SiC-based IGBTs have great theoretical advantages compared with other high-voltage devices, poor wafer quality and immature manufacturing technology hinder the commercialization of SiC-based IGBTs [7]. Therefore, SiC-based IGBT modules are still in the research and development stage. Compared with pure silicon IGBT modules, hybrid IGBT modules have low cost and low technical requirements. In addition, hybrid IGBT modules have better heat dissipation performance than pure silicon modules. Therefore, hybrid modules are conducive to the transition from silicon IGBT modules to SiC-based IGBT modules.

Currently, IGBT chips can dissipate no more than 10 kW of power and adopt single package parallel operation to obtain the required rated current module. Normally, the heat flux of IGBTs used in HEV is about 100–150 W·cm^−2^. With the increase of current capacity and switching frequency, the heat flux will surely exceed 500 W·cm^−2^ in the future [8]. As to junction temperature, silicon IGBT devices have a limit of 150 °C for 6 kV voltage, whereas wide bandgap semiconductors such as SiC and GaN can work at higher temperatures. Considering structural components, welding materials, reliability and cost, the junction temperature is limited to 175 °C by available packaging technology [9,10]. In medium and high-power applications, traditional silicon IGBTs with low switching frequency and new SiC MOSFETs with high cost present performance and cost problems. SiC hybrid IGBT modules combine advantages on both fronts and are considered the best alternative to the traditional silicon IGBTs. In the future, the semiconductor industry expects IGBT power output to increase as technology evolves [11]. Similarly, the higher requirements for junction temperature mean the heat dissipation technology continues to improve to catch up with the development of the IGBT [12,13].

To solve the junction temperature limitation, the thermal management of the chip, packaging, and module assembly have been greatly improved in the past two decades. Amir Sajjad Bahman et al. [14] proposed the concept of a high-energy-density module cooling system, expounded the conceptual knowledge, and improved methods of IGBT cooling system, and also noted the advantages and disadvantages of different cooling schemes. Salem et al. [15] used the method of actual data comparison and analysis to verify the heat dissipation results of the needle-column cooling structure and another microstructure under different working conditions, and verified that the former structure has the best performance. Zhao et al. [16] innovated a unique cooling medium inflow channel, using the best inflow position to achieve the best heat dissipation effect, and the heat was thus taken away more evenly. Of course, in the follow-up, the position structure of some spoiler needles and the production methods of the heatsink have been explored in detail. Traditional air-cooled heatsinks cannot meet the heat dissipation demand, and water-cooled heatsinks are widely used due to their excellent heat dissipation performance [17,18].

However, there are few studies on the heat dissipation and design of the water-cooled structure of high-voltage power amplifier IGBT modules, let alone hybrid IGBT modules with SiC chips. In this paper, the newly designed high-voltage power amplifier water-cooled heat dissipation structure based on finite element numerical simulation, the fluid characteristics, and heat transfer performance of hybrid IGBT modules are studied through the functions of ANSYS Workbench hydrodynamic calculation, heat transfer calculation, and graph calculation results to provide a reference for the subsequent structural optimization of the heatsink.

## 2. Heat Transfer Calculation of IGBTs

### 2.1. Calculation of Thermal Loss

**Figure 2:**
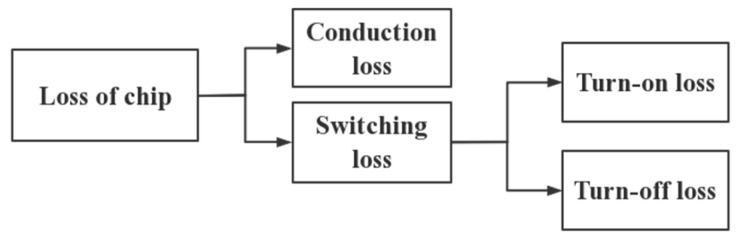
Composition of IGBT module loss.

Thermal management and cooling solutions are an issue of increasing concern for IGBT modules because of their increasing heat loss in a wide range of applications [19,20]. These losses can be further divided into two categories, namely conduction loss and switching loss. For the IGBT module, the thermal loss of the IGBT chips and FWD (free-wheeling diode) chips are mainly considered. The composition of the thermal loss is shown in Figure 2 [21]. The loss of the chip is as follows:

The thermal loss of the IGBT chips is:

(1) $$P_{\mathrm{IGBT}}=P_{sw,I}+P_{c,I}$$

where $P_{sw,I}$ and $P_{c,I}$ represent the switching power loss and the conduction power loss, respectively.

The switching power loss can be expressed as:

(2) $$P_{sw,I}=\frac{1}{\pi }f_{\mathrm{sw}}(E_{\mathrm{on}}+E_{\mathrm{off}})\frac{I}{I_{\mathrm{nom}}}\frac{V_{\mathrm{dc}}}{V_{\mathrm{nom}}}$$

where $f_{\mathrm{sw}}$ is the switching frequency; $V_{\mathrm{nom}}$ is the rated voltage; $I_{\mathrm{nom}}$ is the rated current; $E_{\mathrm{on}}$ and $E_{\mathrm{off}}$ are the lost energy during turn-on and turn-off of the IGBT under $I_{\mathrm{nom}}$ and $V_{\mathrm{nom}}$. $V_{\mathrm{dc}}$ is the DC bus voltage.

The conduction loss of the IGBTs can be expressed as:

(3) $$P_{c,I}=\frac{1}{2}\left(\frac{V_{\mathrm{CE}0}I}{\pi }+\frac{rI^{2}}{4}\right)+M\cos\varphi \left(\frac{V_{\mathrm{CE}0}I}{8}+\frac{rI^{2}}{3\pi }\right)$$

where $V_{CE_{0}}$ and r are derived from the linearization of the output properties of the IGBTs:

(4) $$V_{\mathrm{CE}\left(t\right)}=V_{\mathrm{CE}0}+rI\sin\left(\omega t\right)$$

where ω is the angular frequency of the output current wave, M is the modulation index, and φ is the phase difference between the current and voltage waves.

Similarly, the thermal loss of SiC SBD in the power module can be expressed as:

(5) $$P_{\mathrm{Diode}}=P_{\mathrm{sw},D}+P_{c,D}$$

where $P_{sw,D}$ and $P_{c,D}$ represent the switching power loss and the conduction power loss, respectively. Since the turn-on power loss is negligible, the switching power loss can be expressed as:

(6) $$P_{sw,D}=\frac{1}{\pi }f_{\mathrm{sw}}E_{\mathrm{rr}}\frac{I}{I_{\mathrm{nom}}}\frac{V_{\mathrm{dc}}}{V_{\mathrm{nom}}}$$

where $E_{\mathrm{rr}}$ is the reverse recovery energy loss of the diode at the rated current and the rated voltage. The conduction loss of the diodes can be expressed as:

(7) $$P_{c,D}=\frac{1}{2}\left(\frac{{V^{\prime }}_{\mathrm{CE}0}I}{\pi }+\frac{r^{\prime }I^{2}}{4}\right)-M\cos\varphi \left(\frac{{V^{\prime }}_{\mathrm{CE}0}I}{8}+\frac{r^{\prime }I^{2}}{3\pi }\right)$$

where ${V^{\prime }}_{\mathrm{CE}0}$ and $r^{\prime }$ are derived from the linearization of the output properties of the diodes:

(8) $${V^{\prime }}_{\mathrm{CE}\left(t\right)}={V^{\prime }}_{\mathrm{CE}0}+r^{\prime }I\sin\left(\omega t\right)$$

### 2.2. Thermal Resistance Model

The thermal resistance between the chip and the heatsink is actually equal to the sum of the thermal resistance of each layer in series in the heat dissipation path diagram. The thermal resistance and heat capacity of each layer are expressed by the following equation:

(9) $$R_{\mathrm{th}}=\frac{L}{kA}$$

(10) $$C_{\mathrm{th}}=c\rho AL$$

where $R_{\mathrm{th}}$ is the thermal resistance of each layer, L is the thickness of each layer, k is the thermal conductivity, A is the heat dissipation area of each layer, $C_{\mathrm{th}}$ is the heat capacity of the material, c is the specific heat capacity of the material, and ρ is the density of the material.

**Figure 3:**
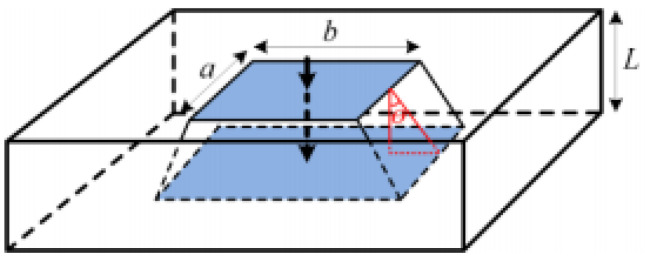
Heat dissipation area on single layer dielectric of IGBT modules.

The thermal diffusion angles from top to bottom layers are not exactly the same, and the thermal diffusion structure of each layer is shown in Figure 3. Due to the different thermal diffusion angle, the heat transfer area is different. In order to accurately calculate the effective heat transfer area of each layer structure, it is necessary to obtain the thermal diffusion angle of each layer through equations as follows:

(11) $$\lambda =\frac{L}{S}$$

(12) $$f\left(\theta \right)=\left\{\begin{array}{c} 5.86\ln\lambda +40.4,\lambda \leq 1\\ 46.45-6.048\cdot \lambda^{-0.969},\lambda \geq 1 \end{array}\right.$$

where S is the effective contact area of adjacent two layers, L is the thickness of each layer, and θ is the thermal diffusion angle, so the heat transfer area can be expressed by the following equation:

(13) $$A=\left(a+2\cdot L\cdot \tan\theta \right)\cdot \left(b+2\cdot L\cdot \tan\theta \right)$$

$R_{\mathrm{th}}$ and $C_{\mathrm{th}}$ affect the steady and dynamic performances of the junction temperature, respectively. When the transient power consumption lasts for more than 0.5 s, it can be considered that the impedance is stable; that is, the temperature difference between the junctions is stable. At this time, the heat capacity of the IGBT can be ignored [22].

**Figure 4:**
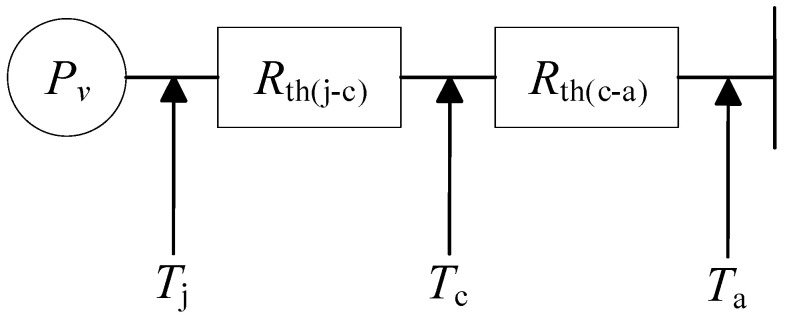
IGBT equivalent computing network for the thermal resistance model.

According to the heat resistance principle, the entire heat dissipation process is simplified into a thermal model network based on a physical model, as shown in Figure 4 [23,24].

According to Figure 4, the coolant temperature and IGBT thermal loss can be calculated for the IGBT and FWD diode, respectively:

(14) $$T_{j,I}=T_{a}+P_{v}\left(R_{\mathrm{th}\left(j-c\right),I}+R_{\mathrm{th}\left(c-a\right),I}\right)$$

(15) $$T_{j,D}=T_{a}+P_{v}\left(R_{\mathrm{th}\left(j-c\right),D}+R_{\mathrm{th}\left(c-a\right),D}\right)$$

where $T_{j,I}$ is the junction temperature of IGBT chips, $T_{j,D}$ is the junction temperature of FWD chips, $T_{a}$ is the temperature of cooling water, $T_{c}$ is the temperature from cooling water to cooling plate housing. It can be seen from Equations (14) and (15) that when the loss is constant, the junction temperature of the chip is affected by the thickness, material, porosity, and other factors of each layer. Therefore, the influence of various factors on heat dissipation is explored from the internal module and heatsink, respectively. This paper discusses the influence of various factors on heat dissipation from the inside of the module and the heatsink.

## 3. Modeling and Solutions of IGBT Modules

### 3.1. IGBT Module Package Structure

**Figure 5:**
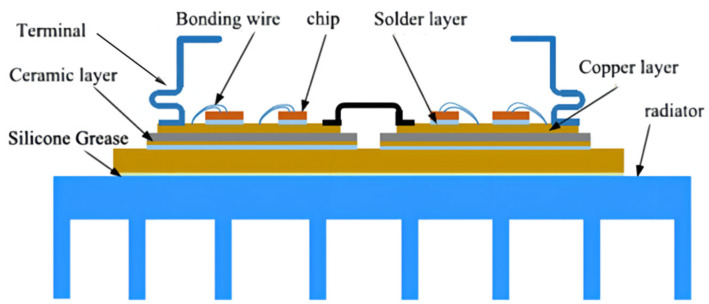
Welded IGBT package structure diagram.

Based on different packaging processes, IGBT modules are mainly divided into two categories: welded IGBTs and press-pack IGBTs. The welded IGBT modules are analyzed with the structure shown in Figure 5.

**Figure 6:**
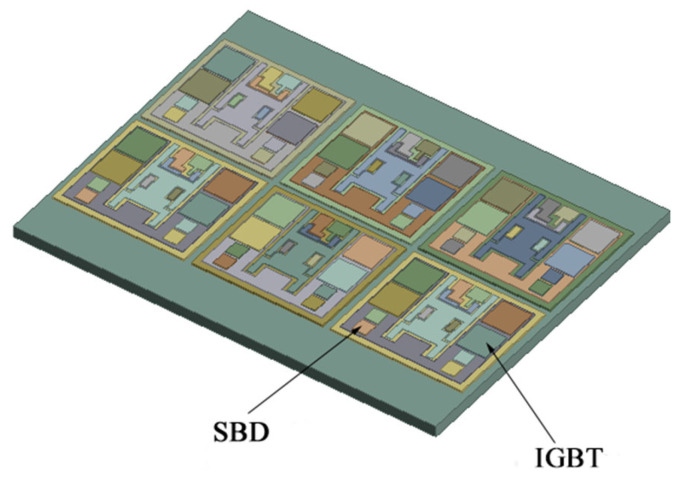
Simulation model diagram of the IGBT module.

**Figure 7:**
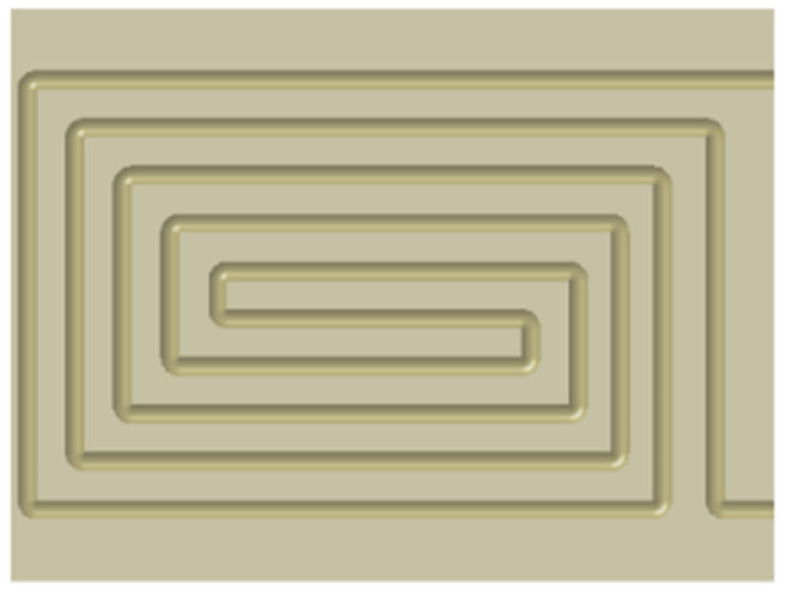
Structure diagram of the water-cooled heatsink.

From top to bottom, IGBT modules can be divided into the chip, chip solder layer, DBC (direct bonded copper) upper copper layer, ceramic layer, DBC lower copper layer), DBC lower solder layer, substrate, and heatsink [25,26]. This research used a 3300 V/1500 high-voltage module with multilayer packaging method, in which the power part was integrated on the substrate through packaging technology. The power module consisted of six modules in parallel, each containing four Si IGBT chips (13.5 × 13.5 mm) and four SiC SBD chips (6.5 × 6.5 mm). Especially for FWD, the SiC SBD (Schottky Barrier Diode) chips replace the traditional Si FRD (fast recovery diode). Every two modules constituted a subsystem, and the rated current of each subsystem was 500 A. A hybrid IGBT module can withstand higher working temperature and greater power shock; some traditional electronic packaging connection methods such as lead solder and lead-free solder cannot meet the needs of high-temperature applications. Due to the high melting point of nano-silver solder, it will not melt during the second welding. Therefore, for research described in this paper, nano-silver solder paste was used as chip solder and 96.5Sn3.0Ag0.5Cu (SAC305) was used as DBC solder. The substrate and heatsink were connected by thermal conductive silicone. The cross-sectional views of the model and the water-cooled structure are shown in Figure 6 and Figure 7.

The geometric model is simplified by considering the complexity of simulation computation. The bonding lines have little effect on the temperature distribution of the power module and they can be ignored when establishing the finite element model. IGBT chips and SBD chips are the heat source of the whole module, and the heat is transmitted down through the solder layer. When dividing the grid, the chip layers and solder layers are fine grids, and standard dimensions are adopted for other parts. The coolant selected was water, with an inlet temperature of 25 °C, and thermal conductivity of 0.648 W·m^−1^·K^−1^. The density is 988.1 Kg·m^−3^, the specific heat is 4.174 J·Kg^−1^·K^−1^. IGBT chip material is Si, SBD chip material is SiC, the substrate material is AlSiC, heatsink material is aluminum alloy, and the specific properties of each part are shown in Table 1.

### 3.2. IGBT Module Boundary Conditions

Temperature field boundary conditions: In simulation calculations, the chips are used as heat sources, and the power losses are directly loaded on the power module to simulate the heat of the chips. When the device is in a long-term state, the total power losses of the module will be composed of the loss of the IGBT chips and the SBD chips. After calculation, the loss of an IGBT chip is 115 W, and the loss of an SBD chip is 20 W. According to the actual work situation, the ambient temperature is 25 °C, and the convective heat transfer coefficient between the surface of the heatsink and the air is 5 W·(m^2^·°C)^−1^.

Flow field boundary conditions: Assuming that the cooling water in the heatsink is continuous incompressible water, its nature is constant; the flow velocity of the cooling water and the pipeline contact boundary is zero. The outlet boundary conditions are designated as fully developed flow, and the inlet temperature is set to 25 °C, the water flow speed is set to 16 L·min^−1^, and the maximum number of iteration steps is set to 300 steps.

## 4. Analysis of Simulation Results

**Figure 8:**
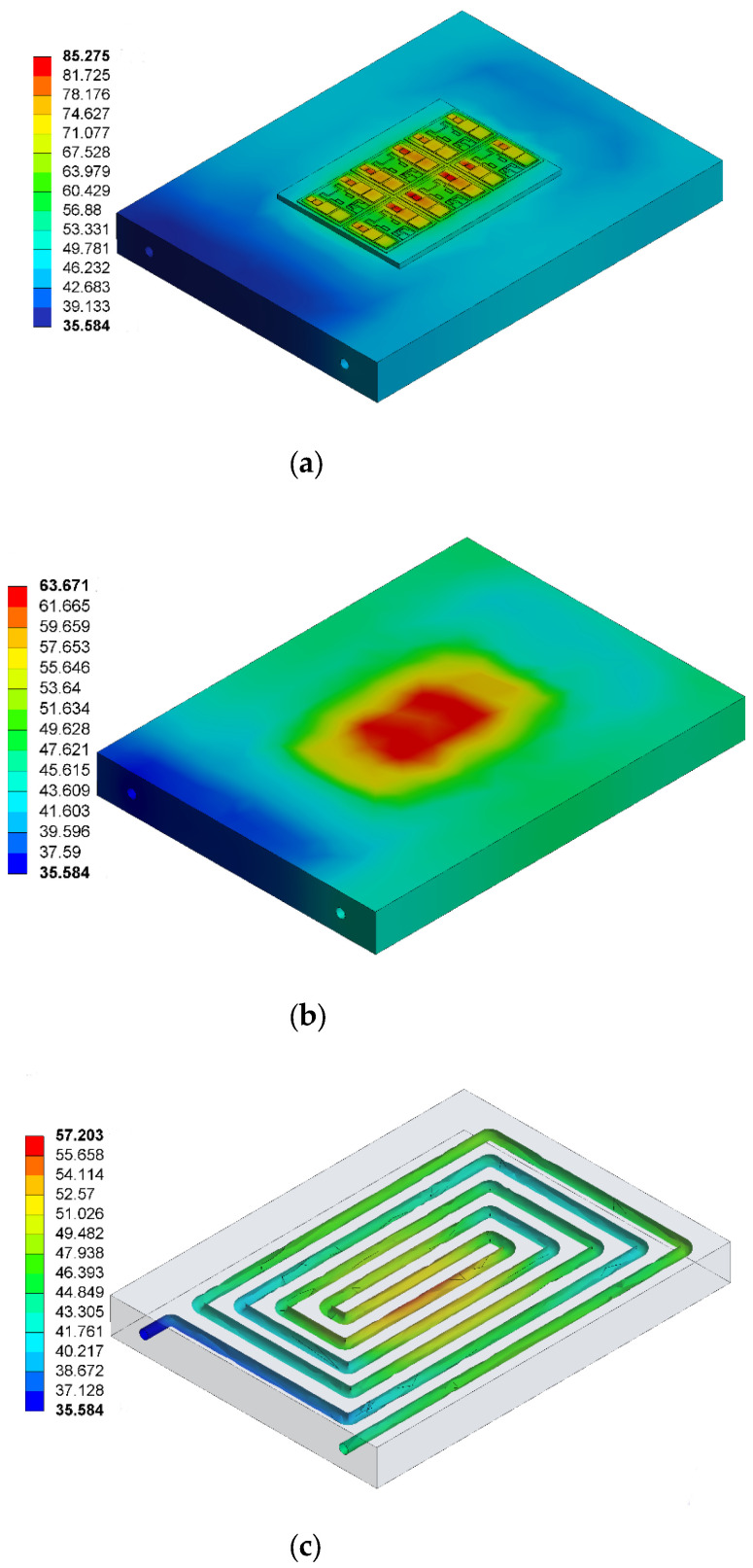
Simulation results (**a**) Overall temperature distribution of the IGBT module, (**b**) Heatsink temperature distribution of the IGBT module, (**c**) Temperature distribution of IGBT water-cooled channel.

After the coupling calculation, the finite element analysis software can obtain the distribution of temperature fields of the IGBT module. The final results are shown in Figure 8.

It can be seen from Figure 8a that as the heat source of the IGBT heat dissipation module, the chips have the highest temperature. The heat emitted by the chip diffuses downward from the bottom of the chip, and the heat dissipation and diffusion area after each layer increase continuously. In addition, the size of SBD chips is smaller than that of IGBT chips, so SBD chips withstand more heat flux than IGBT chips, resulting in them having a higher temperature than IGBT chips; the highest temperature reached 85.275 °C. Figure 8b shows the temperature distribution of the heatsink. It can be seen from the figure that the high temperature of the heatsink is concentrated in the central area of the upper surface, and the maximum temperature is 63.675 °C. The temperature spreads rapidly around, and the temperature difference is obvious. Figure 8c shows the temperature distribution of the water-cooled channel. It can be seen from the figure that the flow temperature increases gradually with the flow and the temperature at the outlet decreases gradually. The flow temperature below the heat source reaches the highest level of 57.203 °C, and the temperature at the outlet is 35.584 °C.

## 5. Factors Influencing the Heat Dissipation

### 5.1. Analysis of Internal Structure

**Figure 9:**
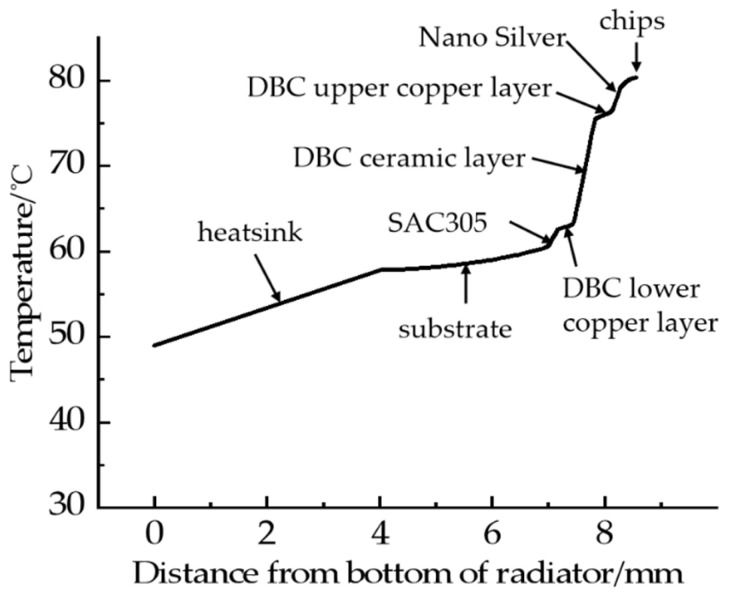
Temperature distribution along the longitudinal direction of the profile.

The material and thickness of each layer in the IGBT module can affect the temperature distribution. The thermal resistance of the IGBT shell is the sum of the junction temperature of each layer in series during the heat dissipation of the IGBT module. Therefore, the influence of the thermal resistance of each layer in the IGBT module on temperature distribution has been studied in detail. The SBD chip is the highest point of the temperature distribution of the whole IGBT module. The vertical direction of the center of the upper surface of the SBD chip is taken as the profile, and the lower surface of the heatsink is taken as the ordinate. From bottom to top, they are heatsink, thermal conductive silicone, substrate, DBC solder layer, DBC lower copper layer, DBC ceramic layer, DBC upper copper layer, chip solder layer, and chip. Figure 9 shows the temperature distribution of the IGBT module along the longitudinal axis.

It can be found from the above figure that the temperature and thickness in the same layer almost show a linear relationship, which is closely related to the thermal conductivity of each layer of materials. The temperature change of materials with small thermal conductivity is relatively large. Due to the use of liquid cooling, the substrate temperature changes only 4.32 °C after 3 mm, reaching 49.2 °C under the heatsink. The most obvious temperature change is in the DBC ceramic layer; only the 0.38 mm thick ceramic layer has a temperature change as high as 14 °C, which is because of the low thermal conductivity of the ceramic layer. In addition to using water-cooled heatsinks instead of air-cooled heatsinks, materials with higher thermal conductivity can also be selected to increase the heat transfer capacity. The thermal conductivity is manifested in the slope of temperature change in the figure. For example, for this paper, nano-silver solder (thermal conductivity of 240 W·m^−1^·K^−1^) was selected as the chip solder layer to replace the traditional SAC305 (thermal conductivity of 32.7 W·m^−1^·K^−1^). In addition, for the DBC ceramic layer, AlN (thermal conductivity of 319 W·m^−1^·K^−1^) was also be used to replace the original Al_2_O_3_ (thermal conductivity of 25 W·m^−1^·K^−1^). The cost of high-thermal-conductivity materials is relatively high, and the industry needs to balance cost and performance according to actual needs.

**Figure 10:**
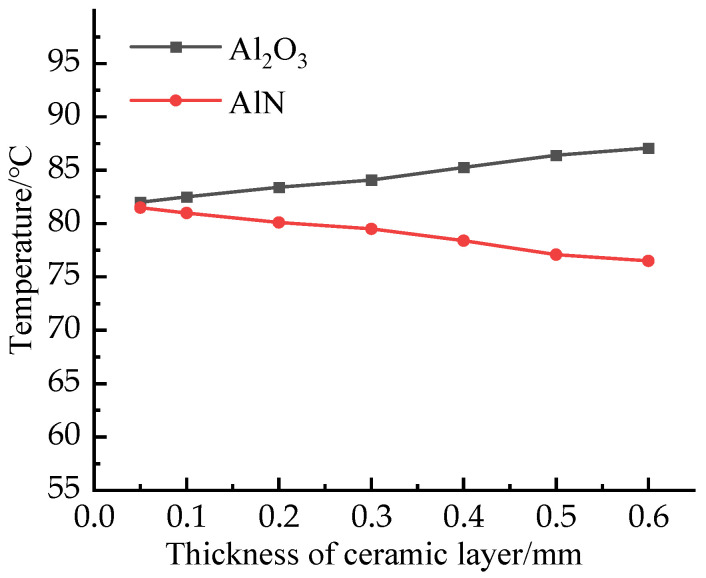
Temperature distribution of IGBT module with the thickness of ceramic layer.

Considering the significant influence of the DBC ceramic layer on IGBT module temperature, the influence of changing the thickness of AlN and Al_2_O_3_ on-chip junction temperature is discussed. The results are shown in Figure 10. Obviously, with the increase of thickness, the chip temperature decreases with the increase of AlN ceramic layer thickness and increases with the increase of Al_2_O_3_ ceramic layer thickness. The influence of the two materials on the chip junction temperature is exactly the opposite. This is due to the high thermal conductivity of AlN and the low thermal conductivity of Al_2_O_3_. It can also be seen from the diagram that with the decrease of the thickness of the DBC ceramic layer, the temperature gap between the two materials is gradually reduced. Therefore, Al_2_O_3_ can be used to save costs within the allowable range of process conditions. Especially for high-power modules with large heat dissipation requirements, if AlN is used as the DBC ceramic layer material, the thickness should be increased as much as possible under the condition of allowable cost. If Al_2_O_3_ is used as the DBC ceramic layer material, the thickness should be reduced as much as possible under the condition of allowable mechanical strength. In addition, the choice of materials should not only rely on thermal conductivity but also consider the impact of various materials on the module.

### 5.2. Analysis of Heatsink Pipe Structure

**Figure 11:**
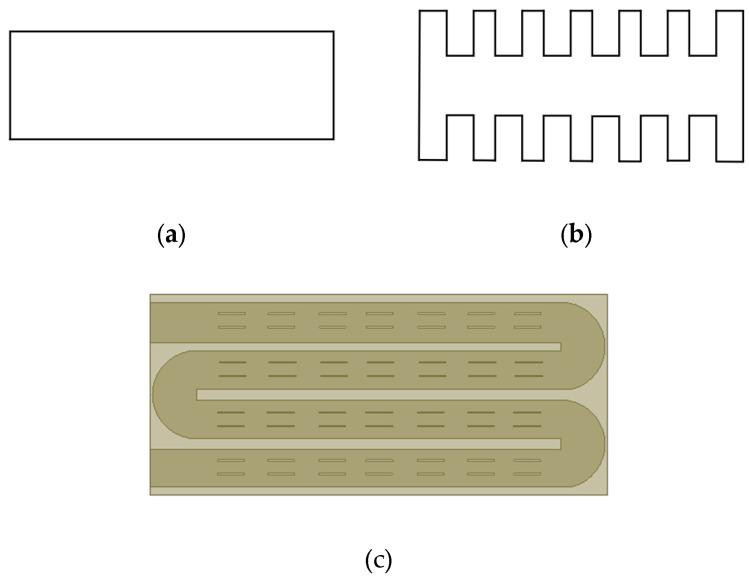
Three different flow channel structures of the heatsink: (**a**) Rectangular structure, (**b**) Concave–convex structure, (**c**) Fin structure.

The different flow channel structures of the heatsink will directly affect the flow state of the fluid in the flow channel and then affect the heat dissipation effect of the heatsink. Therefore, optimizing the design of the pipeline structure can improve the heat dissipation efficiency. When the cross-sectional area of the flow channel is constant, the circular tube is designed as a rectangular structure. It can be seen from the simulation results that the difference between the maximum junction temperature of the IGBT module and the circular structure is not obvious. Therefore, to facilitate the modeling and use of rectangular cooling channels, the structure of rectangular pipes is optimized, and the cross-sectional area of the channels is increased, which indirectly increases the utilization rate of the heatsink. Based on the rectangular heat dissipation channel, the optimization design is carried out. One step is to change the channel section from rectangular to a concave-convex structure, and the other is to add heat dissipation fins in the channel. Figure 11a shows the section of the initial rectangular channel; Figure 11b shows the cross-section of the concave–convex structure flow channel. To ensure the constant cross-section area, the width of the structure is larger than that of the initial structure. Figure 11c shows the top view of the heatsink with spoiler fins. After the design is completed, the fluid–solid coupling temperature is analyzed based on different flow channel structures, and other conditions are kept unchanged to explore the maximum junction temperature of IGBT modules at different flow rates.

**Figure 12:**
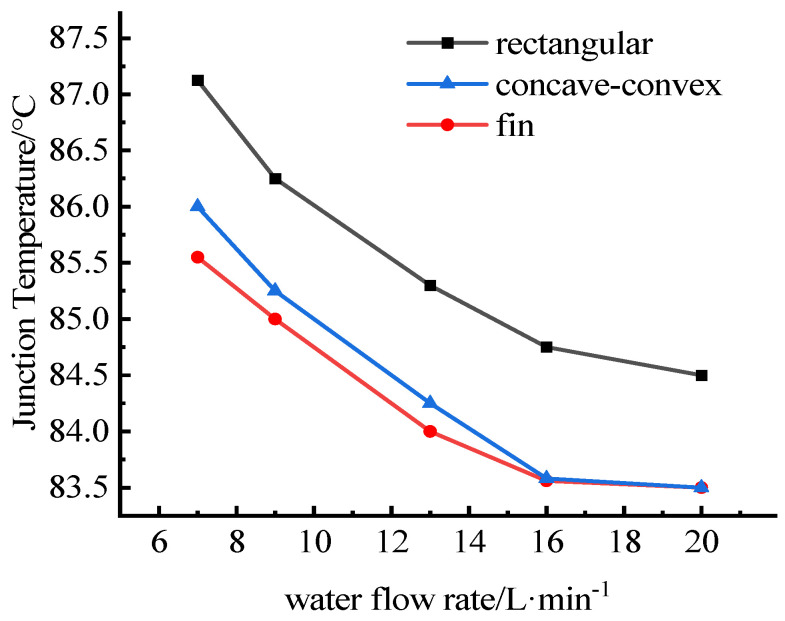
Relations between water flow rate and the junction temperatures of IGBT chips.

**Figure 13:**
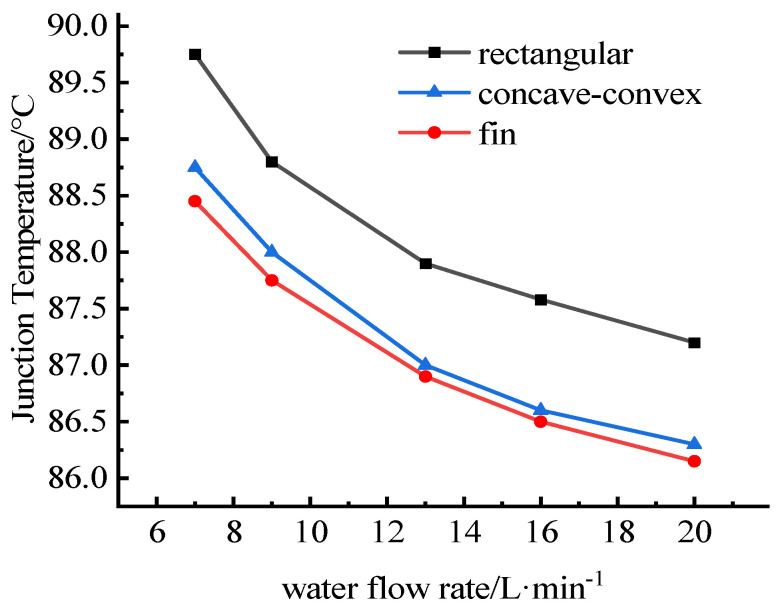
Relations between water flow rate and the junction temperatures of SBD chips.

The relationships between the water flow rate and the junction temperatures are shown in Figure 12 and Figure 13. It can be seen that the junction temperatures of IGBT chips and SBD chips decrease with the increase of cooling water flow. Compared with before optimization, the junction temperature of the optimized heat dissipation channel is significantly lower. The junction temperature difference between models a and b is smaller, and the temperature curves are closer, and with the increase of flow, the temperature curves of concave-convex structure and fin structure are close to coincidence. When the flow rate is the same, the junction temperature of the IGBTs with rectangular flow channel is the highest, while that of the IGBTs with fin flow channel is the lowest.

**Figure 14:**
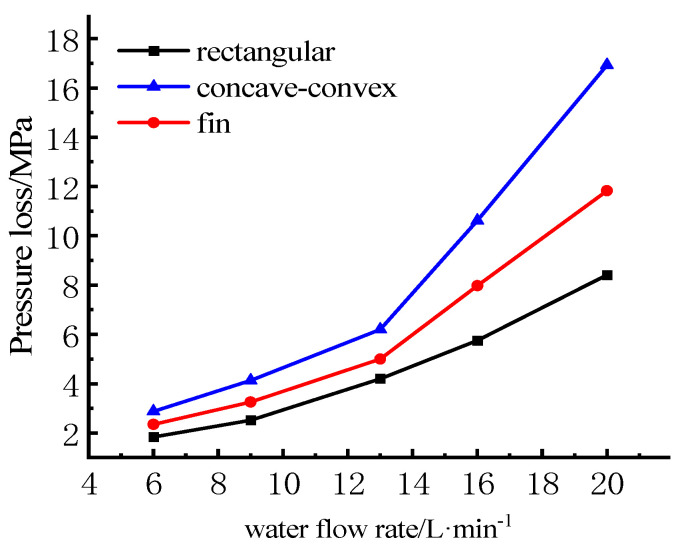
Relations between water flow rate and pressure loss.

The relationships between water flow rate and pressure are shown in Figure 14. It can be seen from the figure that as the water flow rate increases, the pressure loss of each model shows an exponentially increasing relationship. When the coolant flow rate exceeds a certain value, the pressure loss will rise sharply. Compared with rectangular structure, the pressure loss of concave-convex structures and fin structures is larger, which is due to the existence of the fin increasing the spoiler effect. The pressure loss of cooling water will directly affect the energy consumption of the pump. When the pressure loss increases, the energy consumption of the pump will also increase, and the risk of coolant leakage will also increase. Combined with the pressure distribution and junction temperature distribution, considering that the design of concave–convex structures will increase the width of the cross-sectional area so as to increase the overall volume of the module, in addition, compared with other structures, the fin structure has good fluid resistance, so it is undoubtedly a good choice to increase the spoiler fin in the flow channel.

### 5.3. Analysis of Fin Structure

**Figure 15:**
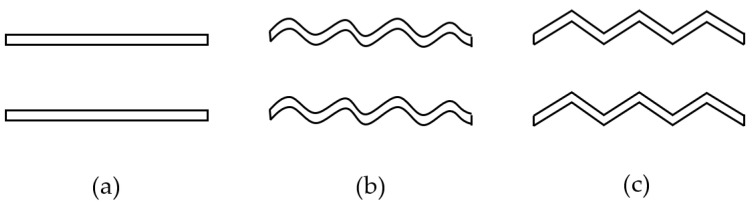
Three different structures: (**a**) Flat fin, (**b**) Sinusoidal corrugated fin, (**c**) Triangular corrugated fin.

From the simulation data in Section 5.2, the maximum junction temperature of the module appears in the position of the SBD chip, and the fin also influences the maximum junction temperature of the module. The three fin structures studied for this paper were a flat fin, a sinusoidal corrugated fin, and a triangular corrugated fin. The fin structures are shown in Figure 15.

**Figure 16:**
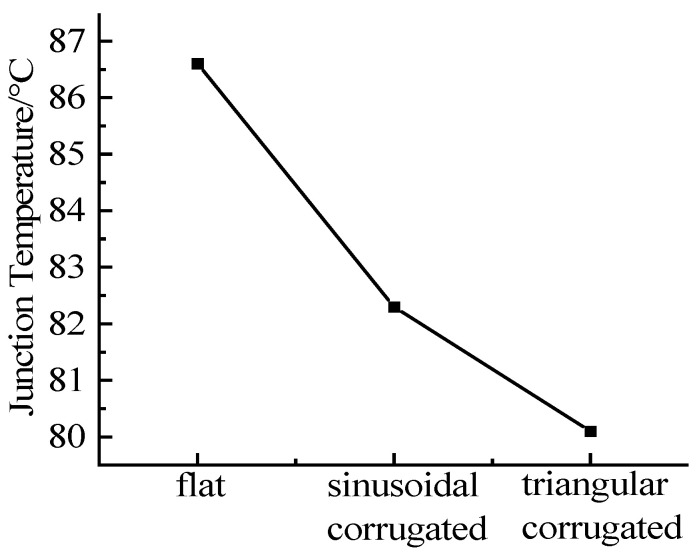
Maximum junction temperature corresponding to three fin structures.

The thickness of the fins was set to 2 mm, the number of fins was 28, the inlet temperature was 25 °C, and the flow rate was 16 L·min^−1^. Except for the structural form of the fins, other boundary conditions were the same. The geometric models of the three fin structures are simulated respectively, and the maximum junction temperatures of the three structures are shown in Figure 16.

It can be seen from the simulation that under the same boundary conditions, the heat dissipation effect of the sinusoidal corrugated fins is the best, followed by that of the triangular corrugated fins, and the straight fins are the worst. The reason is that under the premise of ensuring the same length of the fins, the “folds” of the corrugated fins will increase the convective heat transfer area, and will generate small vortices between the fins, increasing the degree of turbulence, thereby enhancing the heat dissipation effect. The area of the triangular corrugated fins has a relatively small increase, but has little effect on the pressure drop, and is relatively easy to manufacture, which is suitable for small flow heat dissipation.

### 5.4. Analysis of Fin Thickness

**Figure 17:**
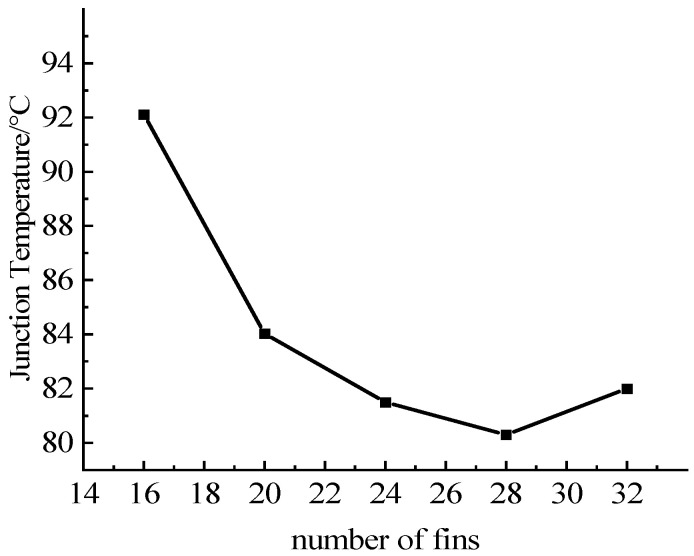
Relationships between the group of fins and junction temperature.

Due to the space constraints of the IGBT module, the number of fins was set from 16 to 32 groups for simulation, the fin structure was sinusoidal corrugated fins, and the thickness of the fins was set to 2 mm. The obtained relationships between the number of fins and the maximum junction temperature of the chips are shown in Figure 17. The data in the table show that when the number of fins increases, the maximum temperature of the IGBT decreases. The reason is that the number of fins decreases and the convection heat transfer area increases, thereby improving the heat dissipation performance. However, an excessive number of fins will increase the pressure drop, and the flow rate will decrease when the flow rate of the water inlet remains unchanged, which in turn will weaken the heat dissipation effect. Therefore, increasing the number of fins must be carried out under the pressure drop requirements.

## 6. Conclusions

To improve the cooling performance of the IGBT module used in motor controllers, the internal thermal resistance and external heat dissipation structure of the module were analyzed, and three flow channel structure models of water-cooled heatsinks are proposed. Through the ANSYS Workbench finite element simulation software, the relationship between the junction temperature of the IGBT module and the pressure drop of the cooling water in the three models within the flow range of 7–20 L·min^−1^ was simulated, and each selected model was tested to verify the effect of the selected model on the cooling effect of the IGBT, and the following conclusions were obtained:

(1) The finite element simulation software was used to perform numerical calculations on the IGBT module and its heat dissipation structure. The simulation results directly reflect the fluid flow and temperature distribution, which can provide a theoretical basis for the optimal design of the heatsink structure.
(2) The result of a numerical calculation based on the coupling field shows that the fluid can take away the heat generated by high-power electronic devices during operation so that the junction temperature of the device during operation is within the allowable range. The use of a water-cooled heatsink can improve the reliability and safety of the device, and the greater the flow rate of the water inlet, the more obvious the heat dissipation effect.
(3) The water-cooled heatsink mainly realizes the heat dissipation of high-power electronic devices through convective heat transfer. The heat transfer area and shape of the heat exchange surface, as well as the fluid medium and flow conditions, are the main factors that affect convective heat transfer. Therefore, at the same flow rate, the junction temperature of the optimized IGBT model is lower than before optimization.
(4) Increasing the contact area between the fins and the water flow can improve the heat dissipation efficiency. Correspondingly, too many fins will increase the pressure drop. When the flow rate of the water inlet is constant, the internal flow rate will decrease, which in turn will reduce the heat dissipation effect. Therefore, a reasonable distribution of fins is very important.

## Figures and Tables

**Table 1:** Material parameters for each component in the IGBT package.

-	Materials	Specific Heat Capacity (J·Kg^−1^·K^−1^)	Thermal Conductivity (W·m^−1^·K^−1^)	Density (Kg·m^−3^)
IGBT	Si	700	144	2330
SBD	SiC	800	370	3200
Upper solder layer	Nano Silver	234	240	8580
DBC	Cu	390	390	8960
	Al_2_O_3_	880	25	3800
	Cu	390	390	8960
Lower solder layer	SAC305	150	32.7	7500
Substrate	AlSiC	760	200	2960
